# Development of an electrolysis based system to continuously recover magnesium from seawater

**DOI:** 10.1016/j.heliyon.2018.e00923

**Published:** 2018-11-17

**Authors:** Yoshihiko Sano, YiJia Hao, Fujio Kuwahara

**Affiliations:** Department of Mechanical Engineering, Shizuoka University, 3-5-1 Johoku, Naka-ku, Hamamatsu, 432-8561, Japan

**Keywords:** Mechanical engineering, Chemical engineering

## Abstract

The continuous resources recovery system utilizing the water electrolysis reaction was developed for recovering magnesium resources from seawater. A set of experiments for forming magnesium hydroxide from the deep-ocean water were carried out at a cathode channel separated by an ion exchange membrane. The ion concentrations of magnesium and calcium in the solution obtained from the outlet of channel were measured by ICP to evaluate the usefulness of the proposed method for the resources recovery system. Moreover, configuration and component in the precipitate formed in the proposed method were analyzed by SEM and EDS respectively. It was found that all magnesium contained in seawater can be precipitated by the proposed method. Moreover, the formation reaction of magnesium hydroxide depends on the quantity of electricity per unit volume of seawater since the production of OH^−^ on the cathode electrode is proportional to the quantity of electricity in the water electrolysis reaction. Subsequently, the effect of deaeration from the deep-ocean water on the purity of magnesium hydroxide was investigated for forming pure magnesium hydroxide. It was found that 99% pure magnesium hydroxide can be created by applying deaeration to the deep-ocean water due to preventing formation of calcium carbonate since the carbon dioxide is removed from the seawater by deaeration.

## 1 Introduction

Magnesium alloys are used as the constructional material since magnesium is a light weight material [1]. Moreover, magnesium has good mechanical characteristics such as high specific strength and high-specific stiffness etc. It is well known that magnesium has a good cutting performance since its cutting resistance is low. Therefore, magnesium is utilized for engineering applications [1, 2, 3]. On the other hand, magnesium batteries using metallic magnesium at an electrode have been investigated as batteries replacing lithium batteries [4, 5]. Industrial magnesium is usually produced from magnesium ore which is mainly distributed in China, Russia, Turkey and some other countries. The 66% of world's production for magnesium compounds is produced in China [6]. However, seawater contains abundant mineral resources. It is well known that concentration of magnesium ion is the second most in all positive ions contained in seawater. Moreover, lithium and strontium ions are also included in seawater.

In the industrial production of mineral from seawater, salt is produced by evaporating seawater concentrated by electrodialysis (ED) which is used for desalination/concentration treatment from ionic solution by using ion exchange membrane [7, 8, 9]. According to W. Zhang, the purity of salt increases by adopting a monovalent ion exchange membrane in the ED system [10]. On the other hand, the effluent treatment for the concentrated seawater drained from reverse osmosis process (RO) has been required in terms of environmental pollution [11]. Under the circumstance, zero discharge desalination technology (ZDD) was proposed for recovery system of drinking water and metal resources, which are combined with RO desalination process and ED process etc. [12, 13, 14, 15]. This technic is expected as the resource recovery method with low cost due to reusing the concentrated seawater drained from RO process.

In industrial production of magnesium from seawater, on the other hand, magnesium hydroxide Mg(OH)_2_ is produced by the reaction with Mg^2+^ ions in seawater and OH^−^ ions contained in some chemicals, namely calcium hydroxide Ca(OH)_2_ or sodium hydroxide NaOH [16]. In some ZDD processes, the method for recovering the magnesium resource consists of the concentration process of seawater by ED and the formation process for the magnesium compound by using chemical [12, 13, 17]. Thus, chemical is needed for creating magnesium compound from seawater in the conventional method. Furthermore, the complicated processes including the pretreatment process are needed to recover high purity magnesium hydroxide. Generally, the batch process with large space and high cost is adopted for magnesium resource recovery. As far as our knowledge, continuous magnesium resources recovery system has never been proposed.

In this study, the continuous resources recovery system utilizing the water electrolysis reaction is proposed for recovering magnesium resources from seawater. A set of experiments for creating magnesium hydroxide from the deep-ocean water were carried out at a cathode channel separated by an ion exchange membrane. The concentrations of Mg^2+^ ion and Ca^2+^ ion in seawater obtained at the outlet of channel were measured by the Inductively Coupled Plasma (ICP) to evaluate the usefulness of the proposed method for the resources recovery system. Moreover, the configuration and component in the precipitate formed in the proposed method were analyzed by Scanning Electron Microscope (SEM) and Energy dispersive X-ray spectrometry (EDS). Substantially, the effect of deaeration from seawater on the purity of magnesium hydroxide was investigated by adopting the deaeration methods of both the acid supplying and the boiling.

## 2 Materials and methods

### 2.1 Materials

**Fig. 1:**
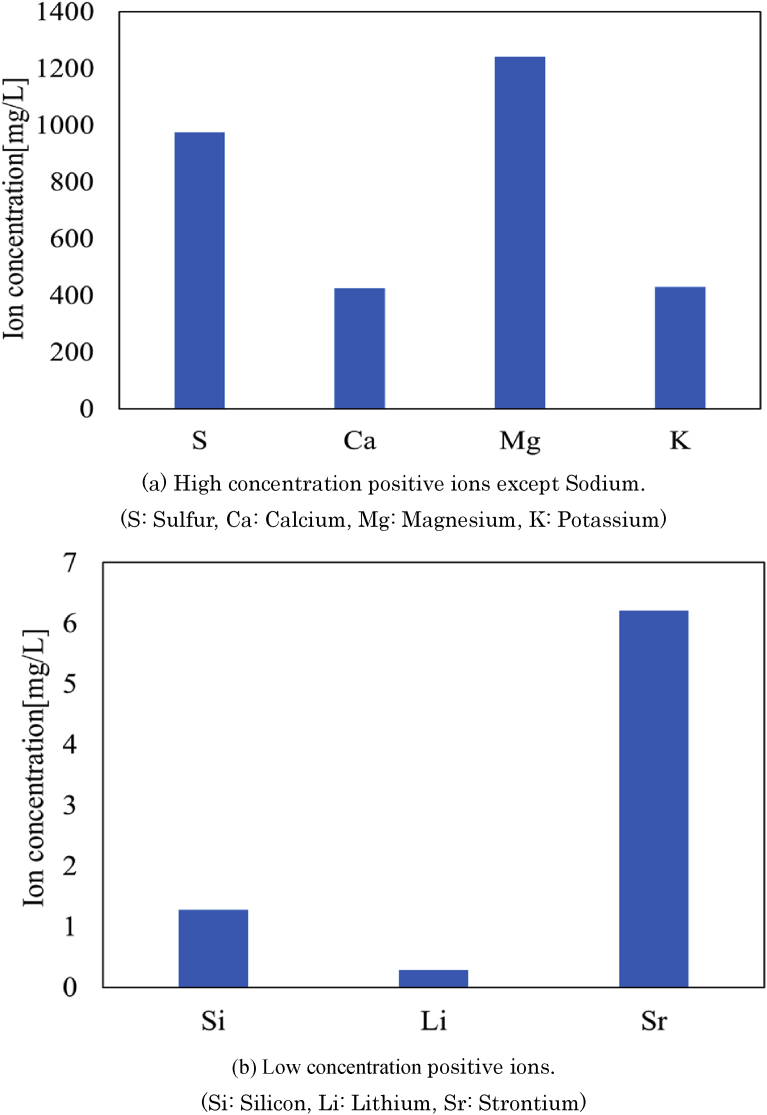
Positive ions concentrations in deep-ocean water obtained from the depth 200 m at Yaizu.

In this study, deep-ocean water obtained from the depth 200 m at Yaizu in Japan was used as experimental solution for magnesium recovery from seawater. Fig. 1 shows ion concentrations at ion components in the deep-ocean water, which were measured by the Inductively Coupled Plasma (ICP, Optima8300, Perkinelmer). Note that sodium concentration is excepted from this figure since its concentration is much higher than the other ions concentration. Therefore, it is found that following sodium, magnesium, calcium, potassium and silicon are mainly included in the deep-ocean water. The concentration of magnesium ion is 1173 mg/L, which is the second most in all positive ions.

**Fig. 2:**
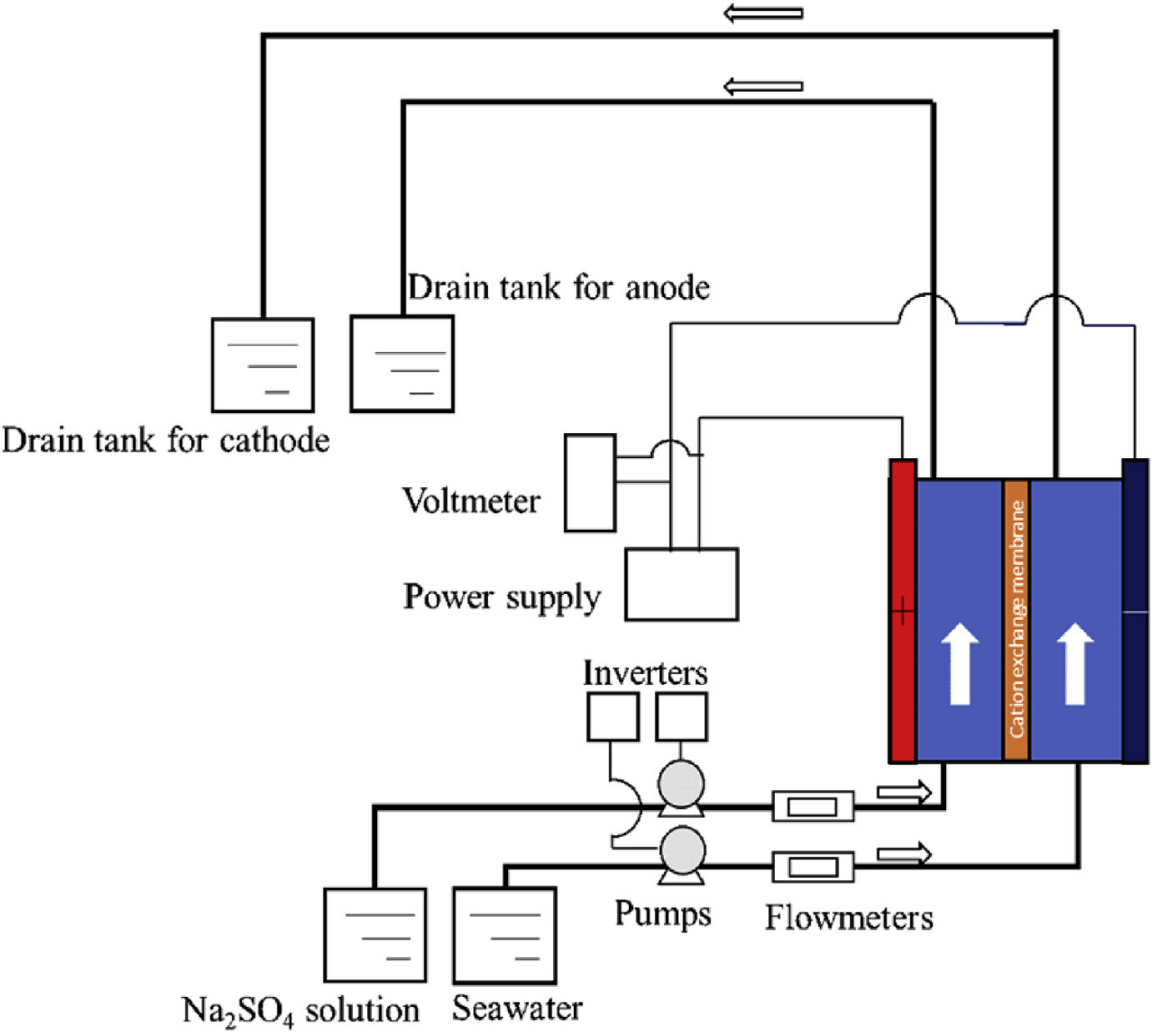
Experiment apparatus for the continuous resources recovery system.

An experimental apparatus is illustrated in Fig. 2, which consists of a filter press electro dialysis stack, a power supply device (GEO142938, GWinstek) and tanks for 5% sodium sulfate aqueous solution Na_2_SO_4_ (Wako Pure Chemical Industries) and deep-ocean water. This deep-ocean water (20 °C) was fed into the cathode channel, while Na_2_SO_4_ solution (20 °C) was fed into the anode channel. Both flow rates were controlled by individual inverters for pumps (MG204XPD17-10S, MAGPON GEAR) and flowmeters (GL200A, GRAPHTEC). Note that each solution passed through the test section only once. Na_2_SO_4_ solution was adopted not to generate Chlorine gas Cl_2_ in this study.

**Fig. 3:**
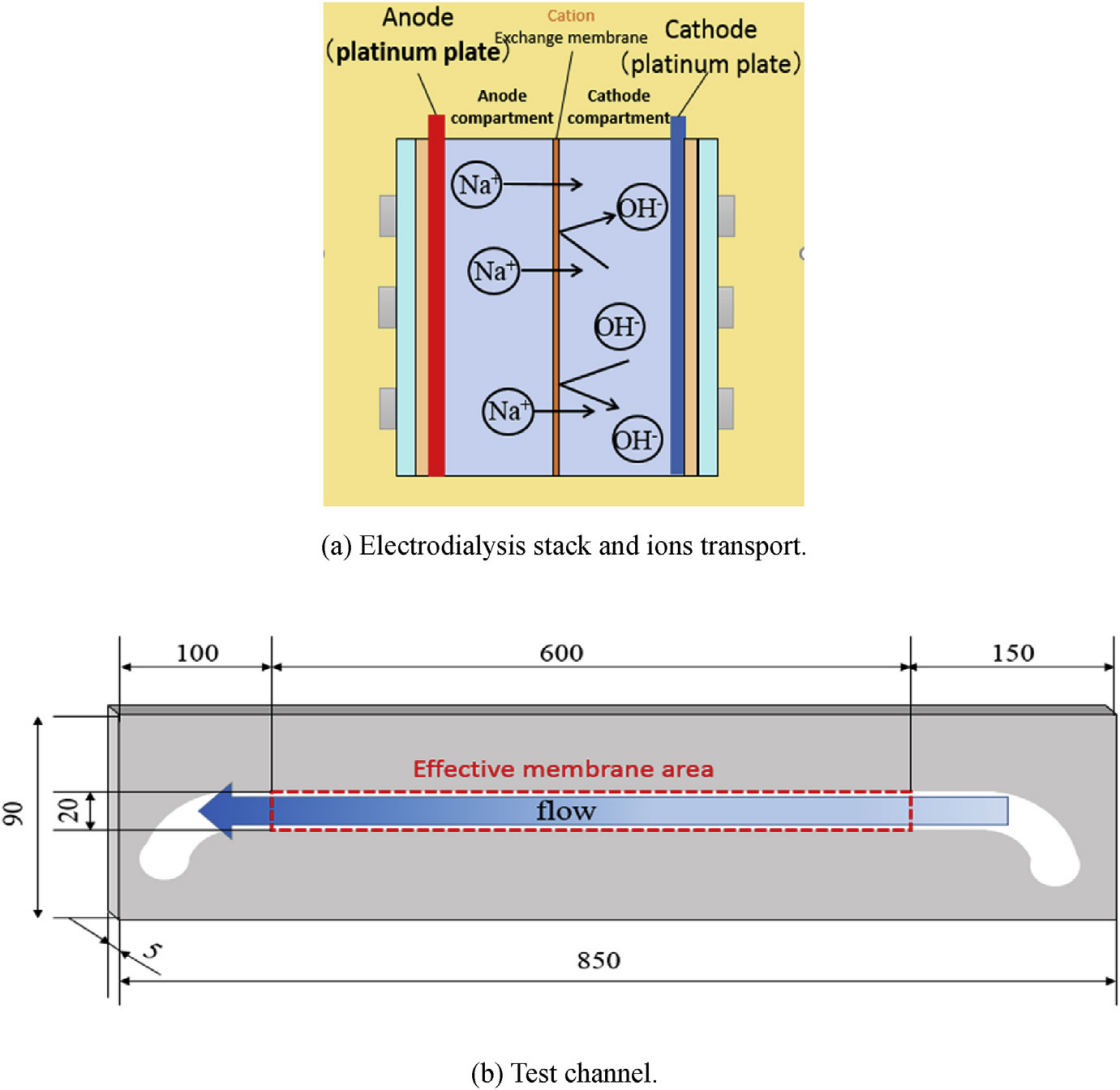
Test section for the continuous resources recovery system.

The inside electrodialysis stack is illustrated in Fig. 3(a). In this study, a cation exchange membrane CMB (ASTOM, Japan) was placed in the middle of channels by pressing with silicone rubbers. The detailed specification for the CMB membrane provided by manufacturer is tabled in Table 1. Moreover, platinized titanium plates (L600mm × H5mm × W20mm, DENBOH) were adopted as electrode plates, which were installed at both channels. When applying the electric current between the electrode plates in both anode and cathode, electrolysis reaction takes place on the electrode plates. In this study, OH^−^ ions generated by water electrolysis are utilized for creating magnesium hydroxide. Therefore, a cation exchange membrane was adopted to prevent the OH^-^ ions generated at cathode from moving to anode. Moreover, this cation exchange membrane plays an important role not to transport Cl^-^ ions from cathode to anode. On the other hand, positive ions penetrate through the cation exchange membrane. The molarity affects the ratio of ions passing through the ion change membrane. In this study, the concentration of Na_2_SO_4_ solution was preliminarily determined to be sufficient high concentration for preventing neutralization by H^+^ ions transported from anode to cathode, by measuring the pH value in the solution obtained at outlet in this experiment.

**Table 1:** Specification of CMB membrane.

Electric resistance	Ω·cm^2^	4.5
Brust strength	Mpa	≥0.40
Thickness	mm	0.21
Recommmend Temperature	°C	≤60
Recommmend pH		0–14

Note that this device is different from conventional ED system. Generally, the conventional ED stack consists a lot pairs of dilute and concentrate compartments by alternately arranged cation exchange membranes and anion exchange membranes. Moreover, a pair of electrode components is placed at both ends of electro-dialysis stack. However, the present stack consists of one cation membrane. In this study, magnesium hydroxide is created by reacting OH^-^ ions generated at cathode and Mg^2+^ ions in the seawater fed directly at cathode. Therefore, membrane is not used for desalination/concentration, so that it is not necessary to use many membranes.

The size of effective membrane area, which is a region contributing to pass the current, was set to same size of electrodes (i.e. L600mm × H5mm × W20mm) by adjusting the size of silicone rubbers for holding the membrane, as shown in Fig. 3(b). A filter press electro dialysis stack was placed perpendicular to the ground. Each solution was fed to the reverse direction of the gravity so as to prevent the root clogging by the formation in the channel. In this experiment, the root clogging was never observed in any parts of the test section.

### 2.2 Chemical reactions

When applying the electric current between the electrode plates in both anode and cathode, the following electrolysis reactions may take place on the electrode plates:

Anode side:

(1) H_2_O → 2H^+^ + (1/2)O_2_ + 2e^-^

(2) 2H^+^ + SO_4_^2-^ → H_2_SO_4_

Cathode side:

(3) H_2_O + e^-^ → (1/2)H_2_ + OH^-^

H^+^ ions are generated on the anode plate, while OH^-^ ions are generated on the cathode plate under the reactions of water electrolysis. Moreover, positive ions are transported through a cation exchange membrane from anode to cathode solution under the influence of the electrical potential difference. In this study, Na^+^ ions primarily move to cathode channel since concentration of sodium sulfate aqueous solution is sufficiently high as compared with H^+^ ion concentration. Current density was set not to reach the limiting current density so that water dissociation does not take place on a cation exchange membrane [18, 19]. Thus, neutralization does not take place at cathode channel since H^+^ ions are not transported to cathode channel, on the other hand, OH^-^ ion cannot permeate through the cation membrane. Therefore, the pH value increases at cathode solution, while it decreases at anode solution.

In the present method, OH^-^ ions generated by water electrolysis are utilized for forming magnesium hydroxide. The solubility of magnesium hydroxide Mg(OH)_2_ is 1.8 × 10^−11^ *mol*/*L* (25 °C), while the calcium hydroxide Ca(OH)_2_ is 5.5 × 10^−6^ *mol*/*L* (25 °C) [20]. When applying the electric current between the electrode plates in both anode and cathode, OH^-^ ions primarily react with magnesium ions rather than Ca^+2^ ions as follows;

(4) Mg^2+^ + 2OH^-^→Mg(OH)_2_

**Fig. 4:**
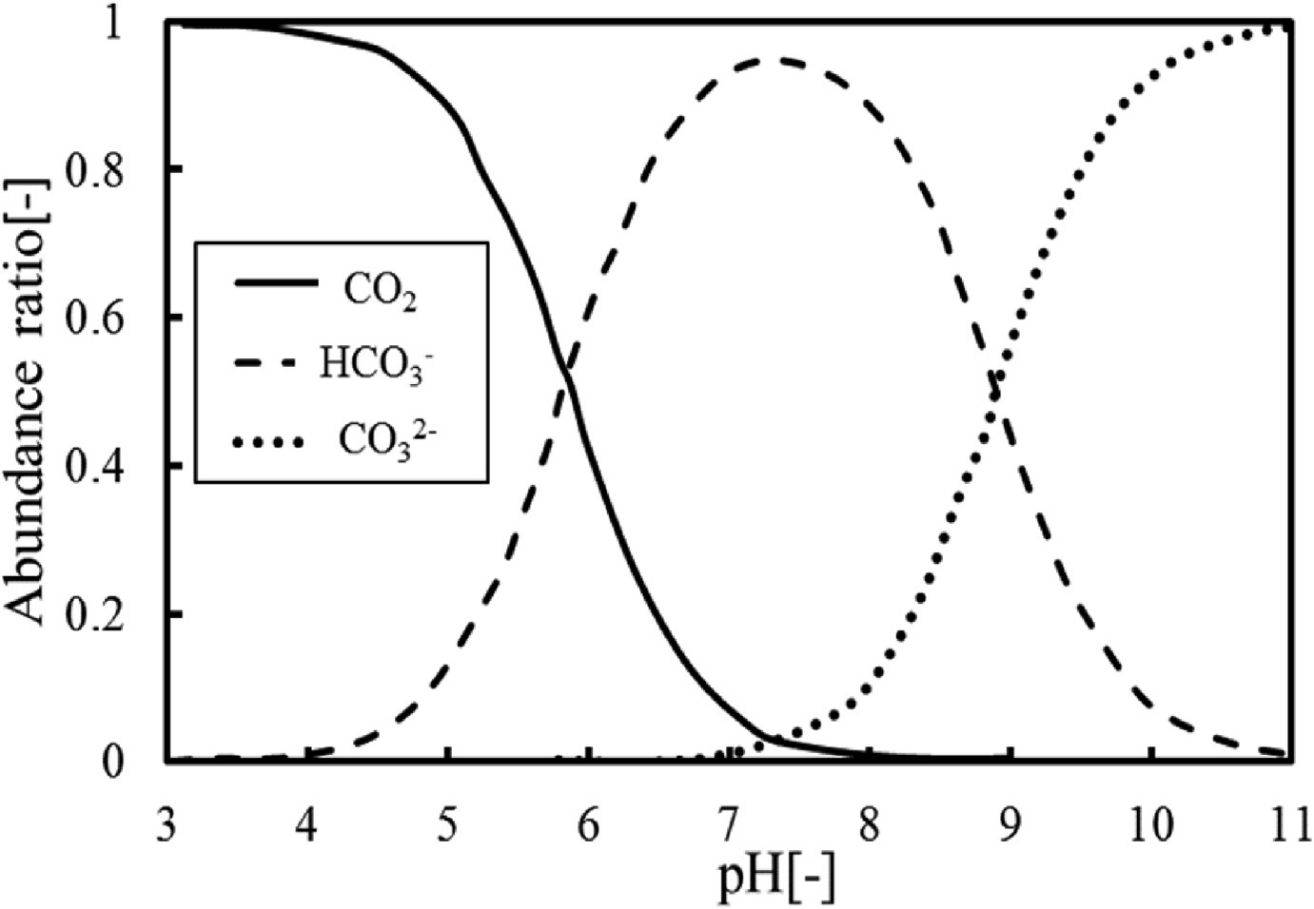
Abundance ratios of carbonic ions against pH value. (Solid line: CO_2_, Dashed line: HCO_3_^-^, Dotted line: CO_3_^2-^).

Thus, magnesium hydroxide can be formed by water electrolysis reaction. However, OH^−^ ions may react with other ions in seawater. Especially, calcium carbonate CaCO_3_ with water insoluble may be created from the viewpoint of solubility product (3.36 × 10^−9^ (mol/L)^2^ [20]). Fig. 4 indicates that the abundance ratios of carbonate CO_2_ (i.e. CO_3_^-2^, HCO_3_^-^ and CO_2_) against the pH value in ionic solutions. The abundance ratios depend on the pH value of solution. The pH value of the deep-ocean water was 7.9, which means that the abundance ratio of bicarbonate ion HCO_3_^-^ is high in the deep-ocean water. However, the pH value increases in the cathode solution by water electrolysis reaction. The bicarbonate ions HCO_3_^-^ may react with OH^−^ ions, so that carbonate ions are generated in the cathode solution.

(5) HCO_3_^-^ + OH^-^→H_2_O + CO_3_^2-^

Moreover, the carbonate ions CO_3_^2-^ may react with calcium ions Ca^2+^ so that calcium carbonate precipitates as follows.

(6) Ca^2+^ + CO_3_^2-^→CaCO_3_

Thus, the OH^-^ ions generated on the cathode plate may be used for forming calcium carbonate.

### 2.3 Experimental procedure

In this experiment, the deep-ocean water was fed into the cathode channel, while Na_2_SO_4_ solution was fed into the anode channel. Each flow rate of seawater and Na_2_SO_4_ solution was set to same, the values were varied 20, 40 and 60 ml/min. Note that each solution passed through the test section only once. Water electrolysis was taken place on both anode and cathode electrodes. In this experiment, a set of experiments were carried under the several quantity of electricity per unit volume of seawater (i.e. the value of the current divided by the flow rate of seawater). The quantity of electricity per unit volume of seawater was set to 3600C/L, 7200C/L, 10800C/L and 14400C/L as tabled in Table 2.

**Table 2:** Experimental conditions.

Flow rate [ml/min]	Current [A]	Current density [A/m^2^]	Quantity of electricity per unit volume of seawater [C/L]
20	1.2	100	3600
	2.4	200	7200
	3.6	300	10800
	4.8	400	14400
40	2.4	200	3600
	4.8	400	7200
	7.2	600	10800
	9.6	800	14400
60	3.6	300	3600
	7.2	600	7200
	10.8	900	10800
	14.4	1200	14400

The solution including precipitate was sampled from the outlet of an electrodialysis stack after reaching steady state. Subsequently, the sample was left for 30 min so as to finish chemical reactions completely. The precipitate and solution at the sample were separated by a paper filter (Specification size 240mm, 5C, ADVANTEC). In this experiment, during filtering the precipitate with a filter paper, pure water was supplied to wash out the other ions for recovering the high purity of magnesium hydroxide. Therefore, the compounds with high solubility, such as NaCl, KCl etc., were removed from the precipitate. Finally, the precipitate on a filter was taken and dried.

The image of the dried precipitate was snapped by Scanning Electron Microscope (SEM, TM3030Plus, HITACHI). The contents of elements in the precipitate was analyzed from beryllium Be to uranium U on the periodic table by Energy dispersive X-ray spectrometry (EDS, TM3000 MICSF+, TM3000 XSTREAM2 OXFORD instrument). Moreover, the concentrations of components in the solution obtained from the outlet of the test section were measured by the Inductively Coupled Plasma (ICP, Optima8300, PerkinElmer).

## 3 Results and discussions

**Fig. 5:**
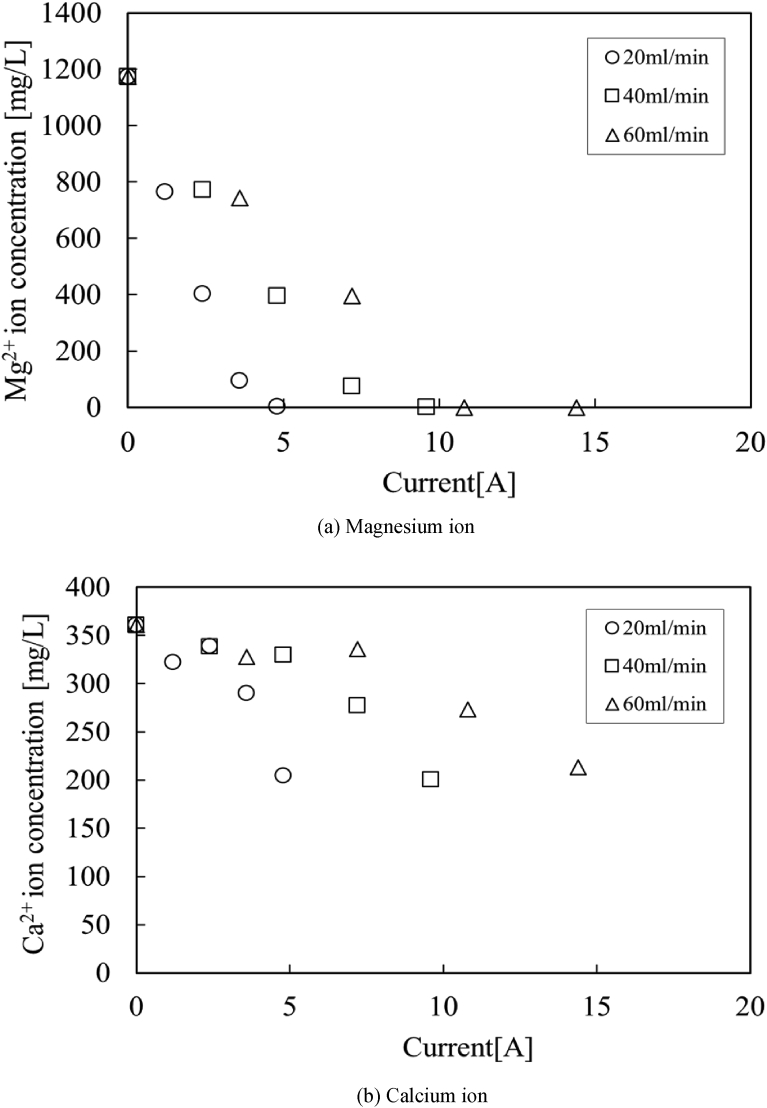
Concentrations in the solution obtained from the outlet. (○: 20 ml/min, □: 40 ml/min, △: 60 ml/min).

Fig. 5 (a) shows the concentrations of Mg^2+^ ions in the solution obtained from the outlet of a test section. The concentrations of Mg^2+^ ions decrease with the electrical current. As can be seen from Fig. 5 (a), all Mg^2+^ ions are completely removed from the deep-ocean water. Therefore, it is found that magnesium can be continually recovered from seawater by the proposed method. The decreases of Mg^2+^ ions are fast under the conditions for low flow rate and high electrical current. On the other hand, Fig. 5 (b) shows the concentrations of Ca^2+^ ions in the solution obtained from the outlet of a test section. It is found that there exist two points removing Ca^2+^ ions from the solution, namely, first is the early time when applying electrical current through the test section, the second is the point in which Mg^2+^ ions deplete from the solution. The decreases of Ca^2+^ ions are also fast in the low flow rate and high electrical current conditions. It indicates that these reactions of removing Mg^2+^ and Ca^2+^ ions from seawater are associated with the quantity of electricity per unit volume of seawater.

**Fig. 6:**
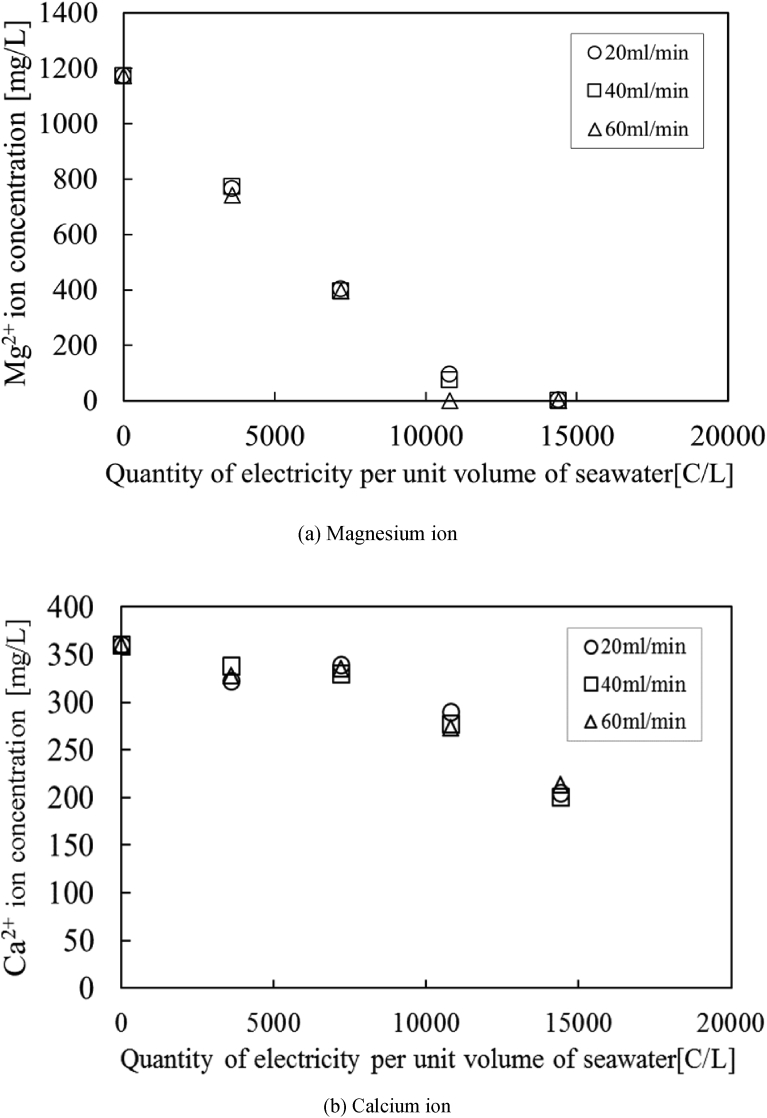
Effect of the quantity of electricity per unit volume of seawater. (○: 20 ml/min, □: 40 ml/min, △: 60 ml/min).

Therefore, Fig. 6 (a) and (b) are redrawn from Fig. 5 (a) and (b) as Mg^2+^ and Ca^2+^ concentrations against the quantity of electricity per unit volume of seawater. All measurement values of Mg^2+^ and Ca^2+^ concentrations are tabled in Table 3. The concentrations are good agreement with each other under the same quantity of electricity per unit volume of seawater. It indicates that the production of OH^−^ ions on the cathode electrode is proportional to the electrical current, so that chemical reactions with Mg^2+^ and Ca^2+^ ions depends on the quantity of electricity per unit volume of seawater. Moreover, it is found that all Mg^2+^ ions are completely removed from the deep-ocean water at the condition of 12,000C/L.

**Table 3:** Mg^2+^ and Ca^2+^concentrations against the quantity of electricity per unit volume of seawater.

Flow rate [ml/min]	Quantity of electricity per unit volume of seawater [C/L]	Mg^2+^concentration [mg/L]	Ca^2+^concentration [mg/L]
Initial values	-	1173.29	360.93
20	3600	764.19	322.22
	7200	402.76	339.13
	10800	94.53	289.83
	14400	3.10	204.80
40	3600	771.86	338.28
	7200	395.72	329.93
	10800	74.46	277.22
	14400	0.17	200.35
60	3600	743.09	327.85
	7200	394.71	335.63
	10800	75.11	273.065
	14400	0.49	213.021

**Fig. 7:**
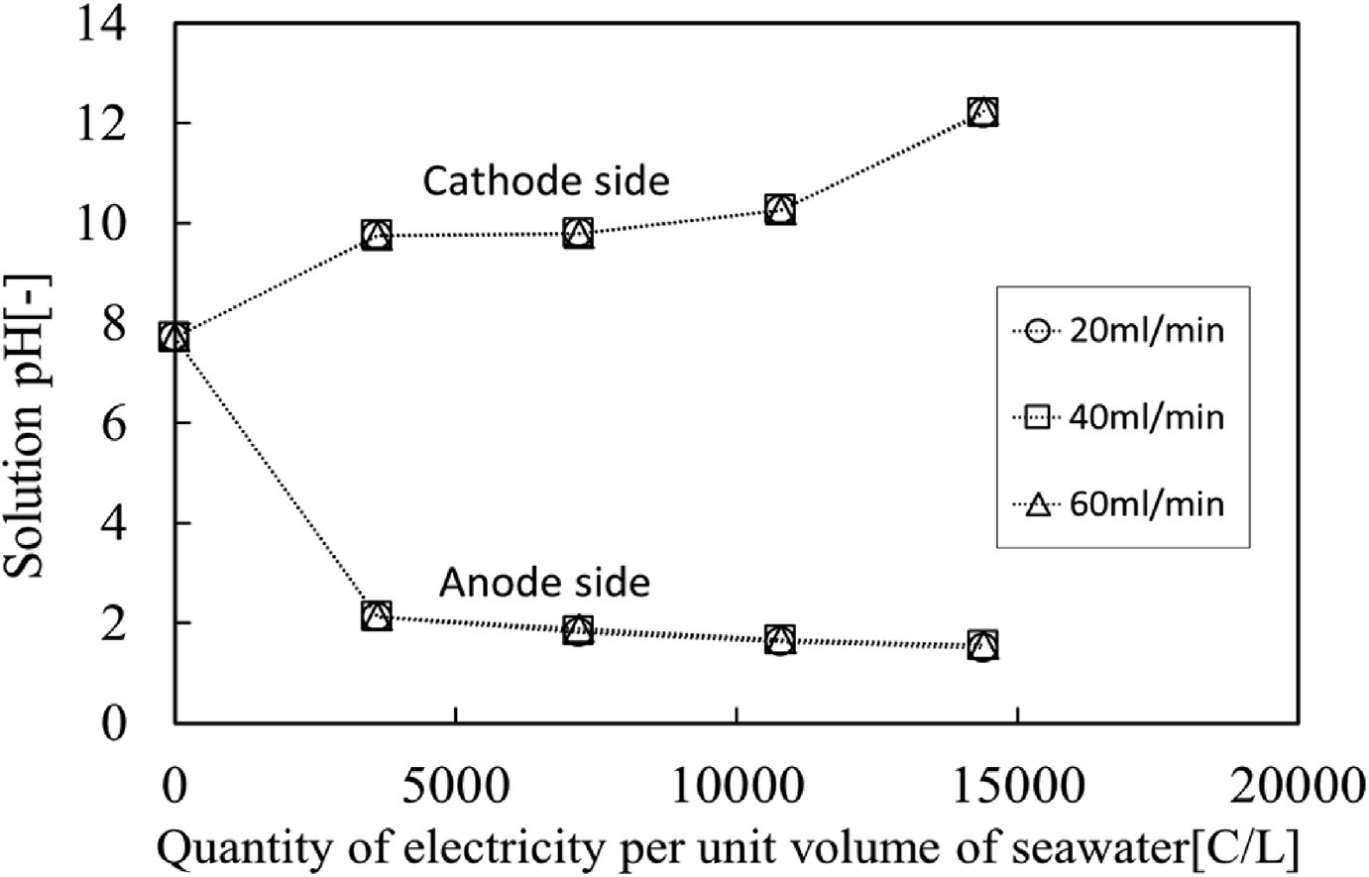
pH values in anode and cathode solution. (○: 20 ml/min, □: 40 ml/min, △: 60 ml/min).

The pH values of anode and cathode solutions are plotted in Fig. 7. H^+^ ions are generated on the anode plate, while OH^-^ ions are generated on the cathode plate by water electrolysis. As can be seen from this figure, the pH value of anode solution decreases with quantity of electricity per unit volume of seawater, while the pH value of cathode solution increases from the initial value 7.9. However, it is found that the pH value of cathode solution increases at low and high regions of the quantity of electricity per unit volume of seawater, on the other hand, the pH value of cathode solution keeps on the 9.8 under the conditions between 3,600 and 10,800 [C/L]. It indicates that OH^-^ ions generated on the cathode plate are used at pH = 9.8 for some chemical reactions with ions in seawater.

**Fig. 8:**
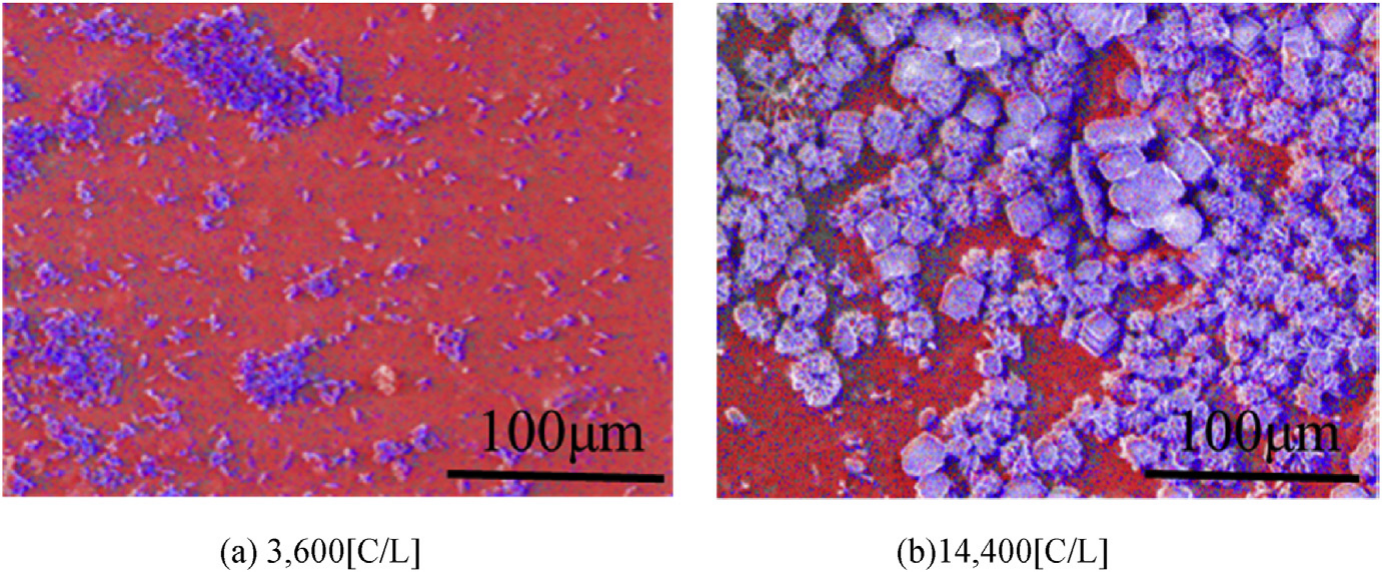
SEM images of precipitate under 40 ml/min (Red: Mg, Blue:Ca).

In order to reveal the components in the precipitate, the element contents in the precipitate was analyzed by SEM and EDS. Fig. 8 shows the images of the precipitate obtained under the conditions 40 ml/min, 3,600 [C/L] and 14,400 [C/L], in which Mg and Ca are taken color mapping to red and blue respectively. As can be seen from Fig. 8, the high magnesium-containing compound is formed in the precipitate obtained by the proposed method. On the other hand, the calcium-containing compound is scattered on the surface of the magnesium compound. Moreover, the calcium formation grows with the electric quantity on the surface of the magnesium formation.

**Fig. 9:**
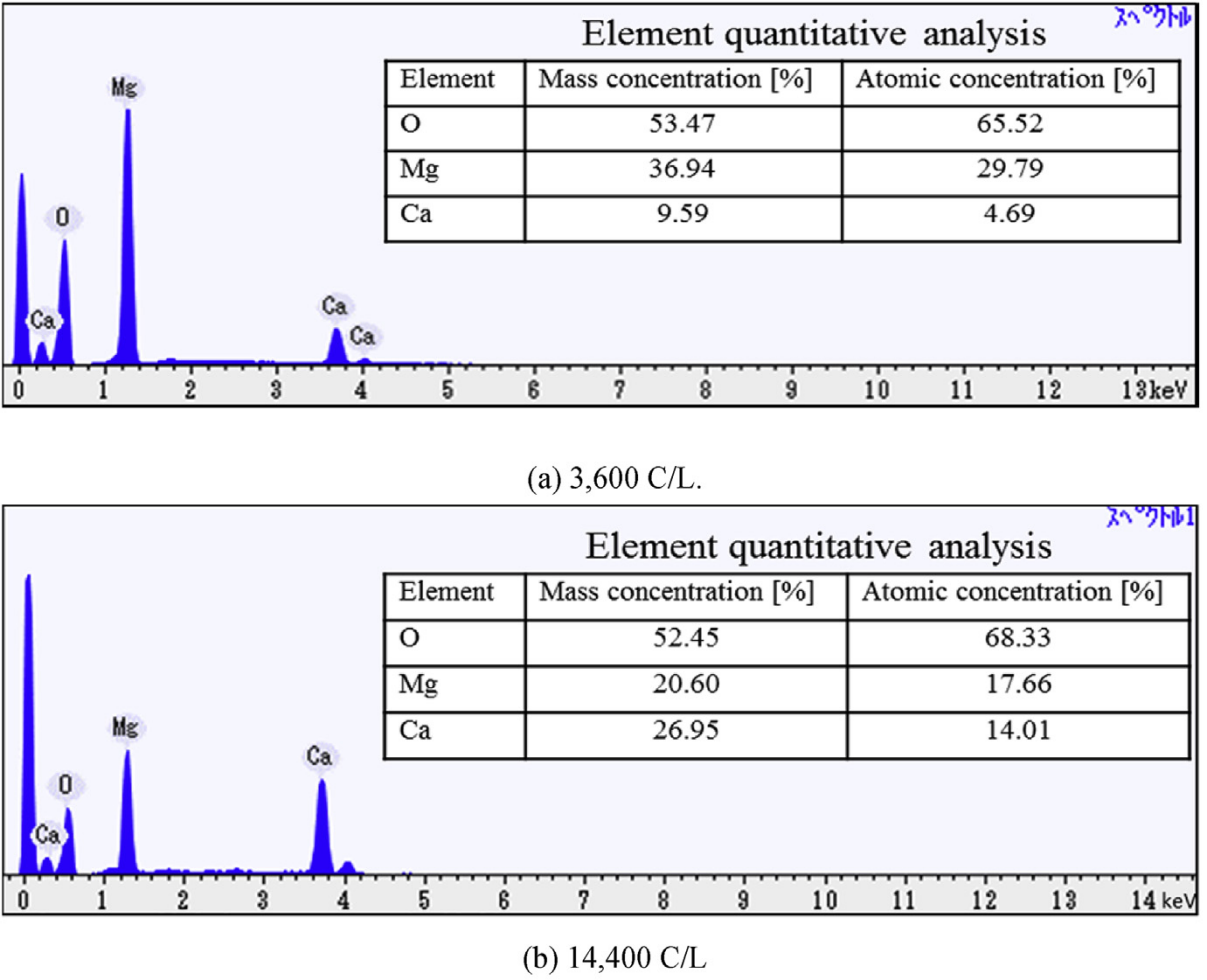
Spectra of precipitate and element quantitative analysis measured by EDS under 40 ml/min.

Moreover, spectra and quantitative analysis results obtained by EDS are shown in Fig. 9, in which the carbon content is not measured since the carbon plate has to be used in the EDS measurement. Note that the values in tables of the quantitative analysis result shows the values excepting carbon content. As can be seen from Fig. 9, only Ca and Mg components as the positive ion are detected by EDS analysis, since the compound with high solubility was washed out with pure water during filtration. Moreover, this compound is mainly composed of magnesium. It can be inferred that the precipitate is magnesium hydroxide since OH^-^ ions are used for the chemical reaction rather than the increase of the pH value in solution. Thus, in the time when the pH value keeps at 9.8 as shown in Fig. 7, magnesium hydroxide is precipitated. Moreover, it is highly probable that the second formation reaction with Ca^2+^ ions would be the reaction for forming calcium hydroxide since calcium formation increases after depletion of Mg^2+^ ions. However, the first formation reaction with Ca^2+^ ions cannot be revealed from these data.

In this study, we want to recovery magnesium hydroxide with high purity. Therefore, these formation reactions with Ca^2+^ ions have to be prevented. It is easy for countermeasure against the second reaction, which is that an appropriate electricity per unit volume of seawater is set so as not to cause the second reaction. However, the first reaction with Ca^2+^ ions would be caused even if we apply an appropriate electricity per unit volume of seawater.

In order to reveal the first formation reaction with Ca^2+^ ions and create pure magnesium hydroxide, the solution deaerated from the deep-ocean water was used in experiments. In this study, two deaeration methods were applied, which are one of the supplying acidum hydrochloricum HCl into the deep-ocean water and the other one of the boiling the deep-ocean water. In the method of the supplying acidum hydrochloricum HCl, the pH value was adjusted to 3.0 by adding acidum hydrochloricum into deep-ocean water. In the method of boiling the deep-ocean water, on the other hand, deep-ocean water was heated at 100 °C for 5 minutes at a pot with a steam-vented lid. The pH value was changed to 7.6 by the boiling process. Each deaerated aqueous solution was used as experimental solution after a lapse of one day.

**Fig. 10:**
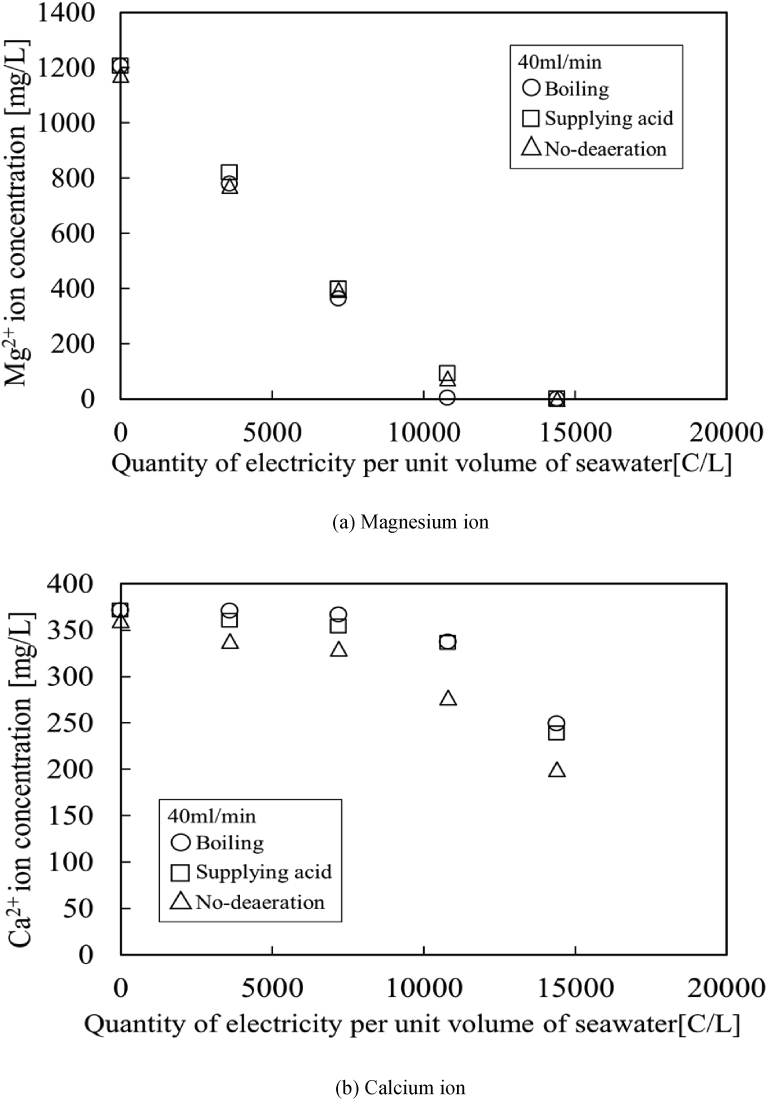
Effect of deaeration on recovering resources from seawater. (○: Boiling, □: Supplying acid, △: No-deaeration under 40 ml/min.)

Fig. 10 shows the concentrations of Mg^2+^ and Ca^2+^ ions in the solution obtained from the outlet of a test section. All measurement values of the present method with the deaerations are tabled in Table 4. It is found that the magnesium formation rate using the deaerated aqueous solution is slightly faster than that of no-deaeration. Moreover, in boiling method, the concentrations of Ca^2+^ ions are kept at an initial concentration until Mg^2+^ ions are depleted from the solution. As can be seen from Fig. 10, the first precipitation with Ca^2+^ ions cannot be observed in the case of boiling aqueous solution. Deaeration would remove carbonate from deep-ocean water. Thus, it is found that the first calcium precipitate is calcium carbonate CaCO_3_, which is reacted with Ca^2+^ ions contained in seawater and the carbonate ions generated by the increase of pH.

**Table 4:** Experimental values of Mg^2+^ and Ca^2+^concentrations under taking deaeration.

	Quantity of electricity per unit volume of seawater [C/L]	Mg^2+^concentration [mg/L]	Ca^2+^concentration [mg/L]
Initical value	-	1173.291	360.9252
Boiling	3600	778.40	370.79
	7200	362.24	366.50
	10800	3.14	337.36
	14400	0.05	248.98
Supplying acid	3600	820.30	360.38
	7200	398.26	354.17
	10800	91.46	336.45
	14400	0.065	238.71

In the case supplying acidum hydrochloricum, on the other hand, calcium concentration is slightly decreased by the formation for calcium carbonate at the early time when applying electrical current, as shown in Fig. 10. As can be seen from Fig. 4, the abundance ratio of carbonic acid CO_2_ becomes high by adding acid into the deep-ocean water. However, some carbonic acid is released to the outside due to its solubility, but not all carbonic acid can be removed even if adding acid. Therefore, carbonate ions regenerated when increasing the pH value of solution by the water electrolysis, so that calcium carbonate is formed by the reaction with Ca^2+^ ions contained in seawater and its carbonate ions.

**Fig. 11:**
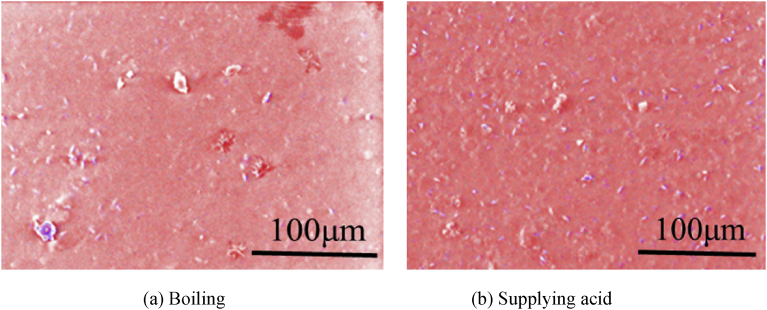
SEM images of precipitate under taking deaeration (40 ml/min, 3,600 [C/L]). (Red: Mg, Blue:Ca).

**Fig. 12:**
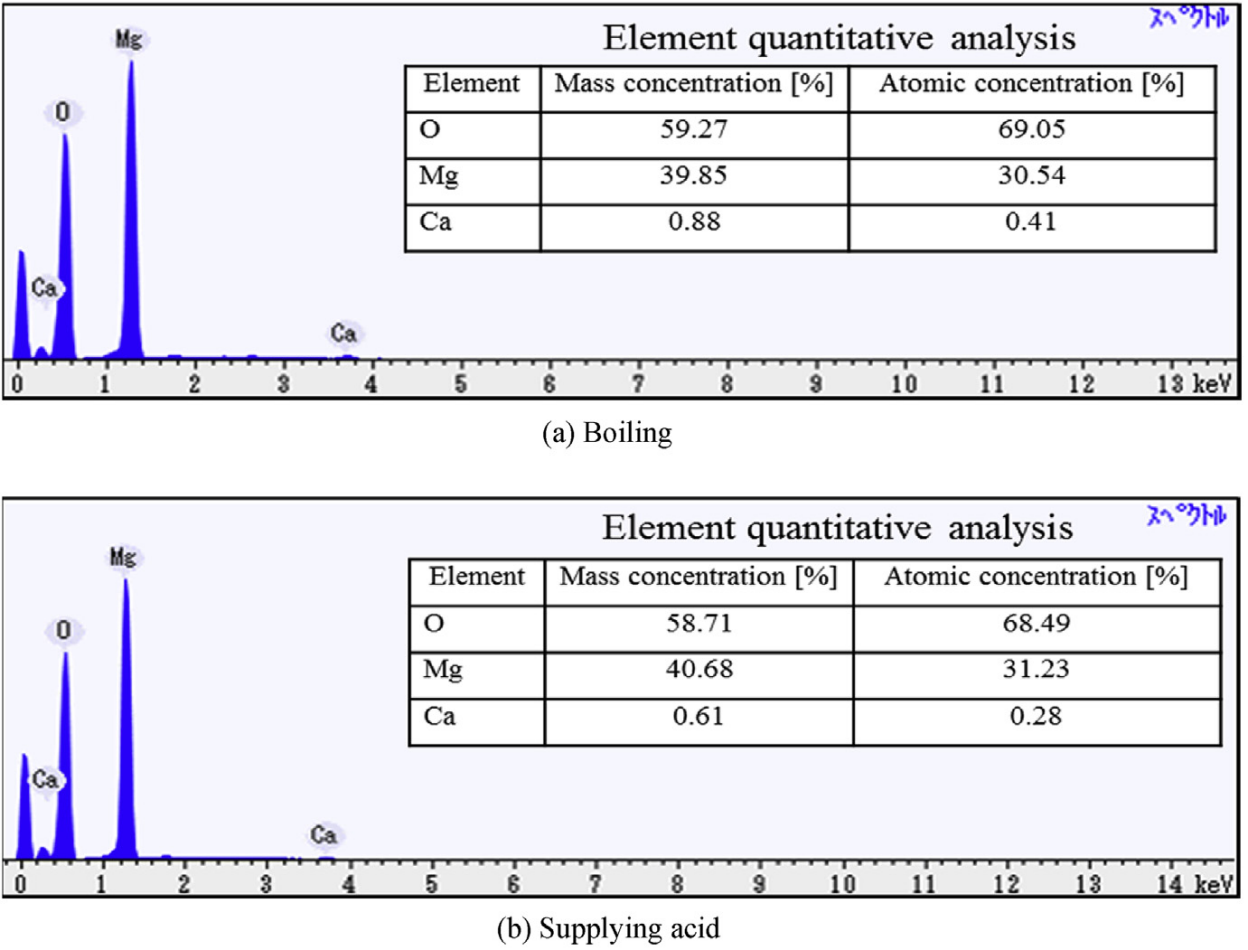
Spectra and element quantitative analysis of precipitate under taking deaeration (40 ml/min, 3,600 [C/L]).

Fig. 11 shows the images of the precipitate obtained in the presented method with the deaerations, in which Mg and Ca are taken color mapping to red and blue respectively. Its experimental condition was set to 40 ml/min, 3,600 [C/L] in order to compare with the case of no-deaeration shown in Fig. 8. As can be seen from Fig. 11, the calcium compound decreases in the precipitates by applying the deaerations as compared with the case of no-deaeration. Moreover, spectra and quantitative analysis results obtained by EDS are shown in Fig. 12. It is found that the magnesium compound is magnesium hydroxide since the molar ratios of Mg and O is almost 1:2 in the precipitates. On the other hand, the molar ratios of Mg and Ca are 30.54: 0.41 in the boiling and 31:23: 0.28 in supplying acidum hydrochloricum, which indicates that the presented method with deaerations can produce 99% pure magnesium compound. Moreover, it is found that calcium carbonate compound does not much affect the purity of magnesium hydroxide in the whole compound in the case of the supplying acidum hydrochloricum. Thus, both deaeration methods are useful to increase the purity of magnesium hydroxide.

Finally, the theoretical cost for producing 1 kg of magnesium hydroxide was calculated to discuss feasibility of the proposed method by comparing with conventional method using chemicals. Note that personnel expenses, equipment fee and maintenance fee etc. are not considered in this study since this research is basic study. In the proposed method, the cost was considered as the fee of electricity for water electrolysis. An experimental condition 40 ml/min (7.2A, 5.58V) was adopted to calculate electric quantity. Moreover, an electricity fee 0.188 USD/kw*h was adopted according to Chubu Electric Power in Japan. On the other hand, in conventional method, the cost was considered as the chemical fee of calcium hydroxide. The fee of calcium hydroxide was adopted as 18.2 USD/kg (Purity 95%, HAYASHI PURE CHEMICAL IND., LTD). Under the above conditions, it was found that costs for producing 1 kg of magnesium hydroxide are 0.97 USD for electricity costs in the proposed method, on the other hand, 14.3 USD for the chemical cost of calcium hydroxide in conventional method. Therefore, it turned out that the presented method is useful from the aspect of cost.

## 4 Conclusions

Continus magnesium resources recovery system from seawater was developed utilizing the water electrolysis reaction. A set of experiment revealed some results as follows;

• All magnesium contained in seawater can be continually recovered at 12,000C/L by the proposed method without any chemical.
• The compound created in the proposed method was found to be magnesium hydroxide by the results of the quantitative elemental analysis by EDS.
• The formation reaction of magnesium hydroxide depends on the quantity of electricity per unit volume of seawater since the production of OH^−^ on the cathode electrode is proportional to the quantity of electricity in the water electrolysis reaction.
• Calcium hydroxide is created after the depletion of Mg^2+^ ions in the solution.
• Calcium carbonate is created at the early time when applying electrical current by the reaction of Ca^2+^ ions contained in seawater and the carbonate ions generated by the increase of pH.
• Deaeration is useful to increase the purity of magnesium hydroxide since it prevents the formation reaction for calcium carbonate. The presented method with deaerations can produce 99% pure magnesium compound
• The theoretical cost for producing 1 kg of magnesium hydroxide is 0.97 USD in the proposed method, which is useful as compared with conventional method.

The feasibility study including all costs for facility is open to discussion. However, according to results obtained in this study, it is concluded that the proposed method utilizing the water electrolysis reaction can be useful for continuous magnesium resources recovery system.

## Declarations

### Author contribution statement

Yoshihiko Sano: Conceived and designed the experiments; Analyzed and interpreted the data; Contributed reagents, materials, analysis tools or data; Wrote the paper.

YiJia Hao: Performed the experiments; Analyzed and interpreted the data.

Fujio Kuwahara: Conceived and designed the experiments; Contributed reagents, materials, analysis tools or data.

### Funding statement

This research did not receive any specific grant from funding agencies in the public, commercial, or not-for-profit sectors.

### Competing interest statement

The authors declare no conflict of interest.

### Additional information

No additional information is available for this paper.
